# The effects of mid-life socioeconomic disadvantage and perceived social support on trajectories of subsequent depressive symptoms among older Taiwanese women

**DOI:** 10.1186/1471-2458-14-384

**Published:** 2014-04-21

**Authors:** Yun-Yu Chen, Chi Chiao, Kate Ksobiech

**Affiliations:** 1 Institute of Health and Welfare Policy, Center for Health and Welfare Policy, School of Medicine, National Yang-Ming University, No. 155, Sec. 2, Li-Nong Street, 112 Taipei, Taiwan; 2 Communication Department, College of Arts and Communication, University of Wisconsin-Whitewater, Whitewater, WI, USA

**Keywords:** Depressive symptoms, Socioeconomic Status (SES), Life-course, Social support, Older women, Taiwan

## Abstract

**Background:**

Scant research has taken a life-course perspective to explore the longitudinal impact of socioeconomic disadvantage and perceived social support on the psychological well-being of older women. We sought to explore whether socioeconomic disadvantage and perceived social support in mid-life are associated with subsequent depressive symptomatology among older Taiwanese women.

**Methods:**

This study was based on data from the Taiwan Longitudinal Study on Aging conducted on a nationally representative sample (n = 1,073) of women aged 50 and above with a 12-year follow up. Mid-life socioeconomic disadvantage was assessed by socioeconomic status (SES) (i.e., educational attainment, major lifetime occupation in adulthood, and employment status) and economic strain. Perceived social support included three dimensions: listening, caring, and sick care. We used the short form of the Center of Epidemiological Studies-Depression (CES-D) scale that measures depressive symptomatology within two domains (negative affect and lack of positive affect). Growth curve models were employed to predict the relationships between mid-life socioeconomic disadvantage, perceived social support, and subsequent depressive trajectories, controlling for aging effects.

**Results:**

Multivariate analyses demonstrated older women in a socioeconomic disadvantaged position are more likely to report higher initial levels of depressive symptoms in both domains; lack of formal education did not exacerbate depressive symptoms in the lack of positive affect domain over time. In addition, mid-life perceived positive social support in caring and sick care was associated with lower initial levels of depressive symptoms in both domains.

**Conclusions:**

Our results suggest independent effects of mid-life socioeconomic disadvantage and perceived social support on subsequent depressive symptomatology among older Taiwanese women.

## Background

Depression is among the most common chronic mental health problems in older Chinese women [1,2], and evidence suggests there are social disparities in symptoms of depression among this population [3,4]. Older Chinese women in a socially disadvantaged position such as lower socioeconomic status (SES) are more likely to suffer from increased levels of depression. These social patterns have been attributed in part to differences in exposure to such socially-based adversities as gender-role socialization [5]. In this regard, women were more likely to be in a lower SES category. Women tended to be economically dependent, more likely to experience stressful life events, and have a limited voice regarding their own mental health needs, all of which may lead to higher levels of depressive symptoms in later life [6].

Longitudinal studies have begun to underscore the importance of life span predictors as contributing factors leading to an increased likelihood of mental health problems [7,8]. This life-course perspective [9] posits that the combination, accumulation, and/or interactions of various personal and professional environments and experiences throughout life synergistically create social disparities and health gradients in later years. This approach emphasizes the importance of cumulative effects from earlier-life to later-life on individual health [9]. The risk clustering model, one of the accumulation approaches, specified negative exposure in an earlier life course leads to subsequent health problems. In turn, such adverse exposure in earlier life may correlate with a wide range of social risks, which impact individual health [9,10]. For example, low midlife SES is associated with more limited social resources, inadequate diet and a steady decline in cognition, all of which may increase the risk of subsequent health problems; as a result, we expected to observe deteriorating effects of accumulating SES disadvantage on subsequent depressive symptomatology [9].

Research has begun to establish the relationship between social disadvantage in early life and one’s health gradient in later life [11,12]. Most depression research to date has sought to examine SES effects on older adults by using a wide range of social disadvantage measures such as educational attainment [13-15], last occupation [14-16], and current income [13,15,17]. Additionally, the stress process model [18] has suggested disadvantaged social groups are more likely than others to exhibit depressive symptoms and other mental health concerns because such groups disproportionately experience a range of adversities, often referred to as perceived stressors, throughout their lives. In this regard, we see economic strain resulting from financial difficulties as yet another important source of stress related to, but independent of, SES [19-22].

A related, but somewhat separate, series of studies has provided evidence coping resources play a buffering role in the relationship between socioeconomic disadvantage and depression [23]. Perceived emotional social support has been shown to have a significant impact on depressive symptoms among older adults [24], in particular among older women [25]. The more individuals perceive the support of family and friends throughout their life events and interactions, the greater their sense of psychological well-being, and the lower their likelihood of exhibiting depressive symptomatology [26,27]. Yet, Antonucci and colleagues [2002] analyzed data gathered from samples of older adults in France, Germany, Japan, and the United States, and their findings suggested perceived social support may not act as a buffering effect on subsequent health. However, that study was based on a regional population, and SES factors in life course were not assessed [7].

The industrialization of Taiwan in the 1960s, with an increase in women’s educational attainment, transformed the labor market with large numbers of women working in factories. This social change disproportionally influenced women in different socioeconomic strata. The risk clustering model hypothesizes that women in lower SES are more deprived of social resources than those in higher SES, which may in turn make them more vulnerable to develop emotional distress in their later years. Surprisingly, scant research has taken a life-course perspective, in particular the risk clustering model, to explore the longitudinal impact of SES as well as perceived social support on depressive symptoms in older women. Although perceived social support has been found to be stable as Taiwanese older adults age [28], little is known about the relationship between mid-life socioeconomic disadvantage, perceived social support, and depressive symptoms in later life, especially in non-western societies [13,29].

To bridge the aforementioned knowledge gap, we hypothesized that mid-life socioeconomic disadvantage and perceived social support are contributing factors to the baseline and growth of depressive symptomatology in later life among older Taiwanese women. The goal of this study was to specifically investigate the cumulative, synergistic effect of mid-life socioeconomic disadvantage and perceived social support on the trajectories of women’s depressive symptomatology from mid- to later life.

## Methods

### Population

**Figure 1:**
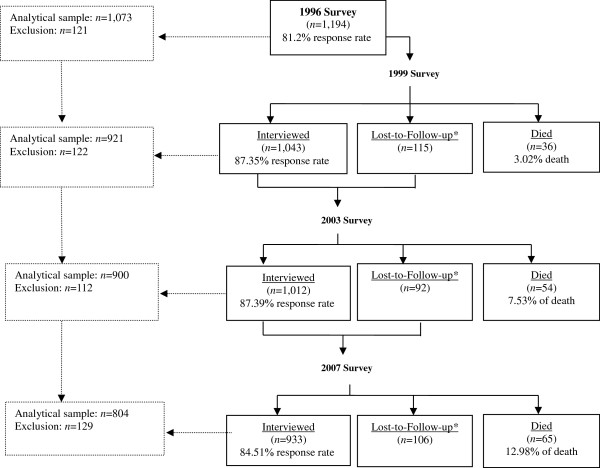
**Female participants in serial surveys in the Taiwan Longitudinal Study on Aging (TLSA) from 1996 to 2007.** Note: LFU is lost-to-follow-up mainly due to moving and rejection to be interviewed.

Data for these analyses were drawn from the Taiwan Longitudinal Study on Aging (TLSA), conducted with a nationally representative cohort sample from 1996 to 2007 in four waves. The survey was designed to study the health and social economic status of Taiwan’s older population, age 50 and above via multi-stage, systematic sampling. The baseline sample included 2,462 male and female participants, with a response rate of 81%. The follow-up surveys were administered in 1999, 2003 and 2007. The response rates were 87%, 87% and 85% for each wave of data collection. We focused on the baseline sample of 1,194 women between the ages of 50 and 67 for this study’s analyses. The sample was further restricted to respondents with complete self-reported data on depressive symptoms and other variables of interest as previously mentioned. These inclusion criteria yielded a cohort sample of 1,073 women in 1996, 921 in 1999, 900 in 2003, and 804 in 2007. The sample flow is shown in Figure 1. The study protocol was approved by the Ethical Committee of National Yang-Ming University.

Study attrition over time was experienced in part due to longitudinal study design and the adult sample. We assessed differences in mid-life socioeconomic disadvantage and social support between continuing participants and those who were lost to follow-up (the results not tabled). The analyses indicated that continuing participants in the fourth wave were significantly more likely to be blue-collars (OR = 1.98, *p* < 0.05) in comparison to the group that was lost to follow-up. The decline in sample size was primarily due to poorer health.

### Outcome and major explanatory measure

Depressive symptoms were measured via a 10-item version of the Center of Epidemiological Studies Depression (CES-D) scale [30]. Each item uses a 4-point scale (0 = no experience to 3 = over 4 days per week) to indicate how often each depressive symptom occurred within the past week. Prior research, and analyses reported herein, indicated two distinct factor domains of depressive symptomatology: negative affect and lack of positive affect [21,31]. More detailed information on psychometric properties of these two domains can be found in Chiao et al. [21]. Among 10 CES-D items, 8 items summed to measure negative affect, generating a score ranging from 0–24 (Cronbach’s α = 0.79-0.85), and 2 items assessed lack of positive affect with a possible range score of 0–6 (Cronbach’s α = 0.85-0.94). Higher scores represented higher levels of depressive symptoms within each domain.

Mid-life socioeconomic disadvantage was indexed by four variables: educational attainment, major adulthood occupation, current employment status in adulthood, and financial strain. Educational attainment was categorized as: no formal education, formal education of 1–6 years, 7–9 years, and 10 years and above. The major adulthood occupation variable had four categories: higher white collar, medium-level occupation (including lower white collar and self-employed), blue collar, and unemployed, as self-reported by each respondent [32]. Employment status had three categories: full-time job, no full-time job, and housekeeper, the last accounting for approximately one-third of the sample in each wave. Economic strain was assessed by asking older women whether they had enough money for living expenses or had experienced a shortage in that regard; responses were categorized as “experienced economic strain” or “have not experienced economic strain” [21].

Perceived social support was measured via three questions [28]. The first was “When you need someone to talk to, how willing are your family, relatives and friends?” Responses to this question, hereafter identified as the “listening” variable, were dichotomized as: positive perception in listening (coded as 1) versus not (coded as 0). The second question was “How caring are your family, relatives and friends toward you?” Responses to this question, hereafter identified as “caring”, were dichotomized as: positive perception in caring versus not (coded as 0). Finally, the third question asked “When you are sick, how reliable are your family, relatives and friends?” Responses for this variable, hereafter referred to as “sick care”, were dichotomized as: positive perception in sick care versus not (coded as 0). The analyses by Cornman et al. [28] further suggested the summation of the three items to produce a social support scale, which ranged from 0 to 3.

### Analytical strategy

All analyses were conducted using STATA 12. We applied growth curve models estimated within a multilevel modeling framework that simultaneously consider changes within and between individuals. These models include two parts: the symptom trajectories within individuals over 4 time points (Level 1) and variation of these trajectories across the entire sample of women (Level 2) [21,33]. That is, these two-level growth curve models were specified with age at Level 1, and nested within individuals at Level 2 to assess mid-life effects of socioeconomic disadvantage and perceived social support on trajectories of depressive symptomatology. In such a growth curve, the intercept (β_0_) and the slope (β_1_) represent the level and change rate of mid-life covariates in depressive symptomatology for a woman. The interaction of the slope with mid-life covariates at Level 2 describes group differences with respect to mid-life SES and social support regarding changes in depressive symptomatology.

We assessed the relationships between socioeconomic disadvantage, perceived social support in mid-life, and changes in depressive symptoms as age increased in different domains separately. Mid-life social disadvantage and its interactions with intercept and slope at Level 1 examined whether there was significant variability in depressive symptoms at the time of initial measurement and over time for different categories of socioeconomic disadvantage. An additional analysis using growth curve modeling was conducted to confirm whether the perception of social support remained stable as age grew as suggested by previous research [28]. We found no effect of age on the trajectories of three measures of perceived social support. Therefore, we concluded baseline social support was a time invariant characteristic in this study.

## Results

For the baseline data in 1996, approximately 45% of the women did not receive a formal education, and 41% of them received only primary education (See Table 1). Furthermore, approximately half reported their adulthood major occupation as being blue collar, with one-third never being employed. About 44% of them engaged in housework, and one-fourth experienced economic strain. About 80% reported positive perceptions of social support on caring and sick care, with 70% reporting positive perceptions on listening. Sample characteristics were similar across four waves of the survey. The level of depressive symptoms in the negative affect domain increased over time, whereas the level of depressive symptoms in the lack of positive affect domain seemed to remain relatively constant (results not shown).

**Table 1:** Sample characteristics at 1996, Taiwan Longitudinal Study on Aging (TLSA)

	**% or mean (SD)**
***Mid-life SES***	
Education attainment	
No formal education	45.29
Formal education	
1-6 years	40.73
7-9 years	8.01
10 years and above	5.96
Adulthood major occupation	
Higher white collar	9.78
Medium-level	9.78
Blue collar	48.89
Unemployed	31.55
Employment status	
Full-time job	27.77
No full-time job	27.96
Housekeeper	44.27
Economic strain	25.26
***Perceived social support***	
Positive perception of social support	
Listening	72.60
Caring	84.90
Sick care	82.20
***Individual characteristics***	
Age	57.74 (4.74)
***Psychological distress***	
Depressive symptomatology	
Negative affect (0–24)	3.59 (4.68)
Lack of positive affect (0–6)	2.01 (2.28)
N	1073

**Figure 2:**
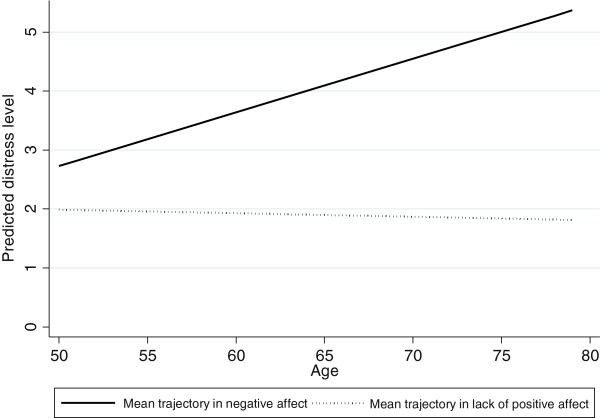
Mean trajectory of depressive symptomatology on negative affect and lack of positive affect without covariates, the Taiwan Longitudinal Study on Aging (TLSA) from 1996 to 2007.

**Figure 3:**
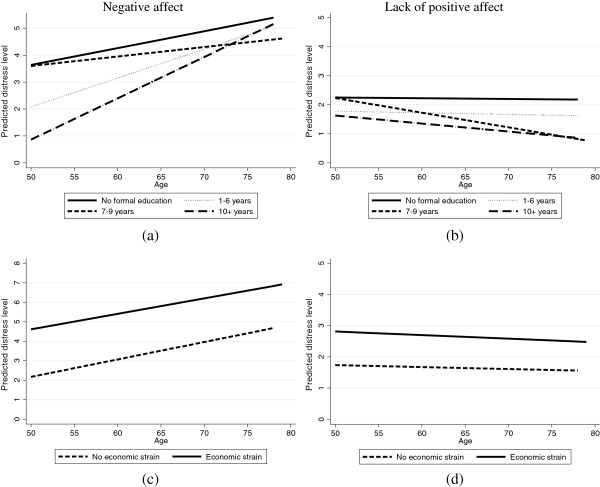
Mean trajectory of depressive symptomatology on negative affect and lack of positive affect by education attainment and economic strain, the Taiwan Longitudinal Study on Aging (TLSA) from 1996 to 2007: (a) negative affect by levels of education attainment; (b) lack of positive affect by levels of education attainment; (c) negative affect by whether women experienced economic strain; (d) lack of positive affect by whether women experienced economic strain.

To illustrate trajectories of depressive symptomatology in growth curve frameworks, we plotted the predicted mean trajectory of depressive symptomatology on negative affect and lack of positive affect. As shown in Figure 2, the predicted trajectories without covariates were plotted separately for two domains of depressive symptomatology. In contrast to the lack of positive affect domain, symptoms of negative affect increased over time as women aged (β_1_ = 0.09, *p* < 0.001). Figure 3 demonstrated the predictive trajectories stratified by education (Figure 3a and Figure 3b) and economic strain (Figure 3c and Figure 3d). As shown in the lack of positive affect domain, differences across education (Figure 3b) and economic strain groups (Figure 3d) were mainly observed at the initial level of the depressive trajectories; gaps in depressive symptomatology clearly increased over time between women with no education and 7–9 years of education. Similar trends were also found in the negative affect domain.

Table 2 presents the results of the growth curve analysis for the negative affect domain and lack of positive affect domain. The coefficients show the net effect of a one-unit change in the independent variable on the level of depressive symptoms. For categorical predictors, the coefficients represent the depressive level for those in a particular category relative to those in the reference. The effects of mid-life predictors on changes over time in depressive symptomatology are indicated by “*mean growth (β_1_)”.

**Table 2:** Maximum likelihood estimates from the growth curve models with random intercept and slope of depressive symptomatology in TLSA

	**Negative affect**		**Lack of positive affect**	
	**Est. (β)**	**S.E.**	**Est. (β)**	**S.E.**
***Mid-life socioeconomics***				
Education attainment (ref = No formal education)				
Formal education				
1-6 years	−1.21***	0.38	−0.36^§^	0.19
7-9 years	0.47	0.64	0.18	0.32
10 years and above	−2.22***	0.68	−0.50	0.34
1-6 years*mean growth (β_1_)	0.04	0.03	−0.002	0.01
7-9 years*mean growth (β_1_)	−0.03	0.05	−0.05*	0.02
10 years + *mean growth (β_1_)	0.09^§^	0.06	−0.02	0.02
Adulthood major occupation (ref = Blue collar)				
Higher white collar	0.21	0.38	0.14	0.15
Medium-level	0.10	0.36	0.06	0.15
Adulthood unemployed	−0.27	0.26	−0.004	0.01
Employment status (ref = Housekeeper)				
Full-time job	−0.001	0.27	0.06	0.11
No full-time job	0.70**	0.25	0.01	0.10
Economic strain	1.98***	0.42	0.89^***^	0.21
Economic strain*mean growth (β_1_)	0.002	0.03	−0.005	0.01
***Life related buffer and stressor***				
Positive perception of social support				
Listening	0.07	0.45	−0.04	0.22
Caring	−1.74**	0.59	−0.67*	0.29
Sick care	−0.94^§^	0.51	−0.63*	0.25
Listening*mean growth (β_1_)	−0.03	0.03	−0.01	0.02
Caring*mean growth (β_1_)	0.10*	0.04	0.02	0.02
Sick care*mean growth (β_1_)	−0.02	0.04	0.02	0.02
Symptom level, intercept (β_0_)	5.07***	0.61	3.07***	0.30
Mean growth rate, linear (β_1_)	0.01	0.04	−0.03	0.02
***Random effects***				
Variance in random intercept	3.15***	1.47	0.93***	0.32
Variance in random linear slope	0.01	0.01	0.001	0.002
Variance in residuals	13.53***	0.43	3.71***	0.11

^§^*p* < 0.1, *p < 0.05; **p < 0.01; ***p < 0.001.

In the negative affect domain, all of the mid-life socioeconomic disadvantage indicators except for adulthood major occupation were significantly associated with the initial status of depressive symptoms. Whereas, only education in the mid-life socioeconomic disadvantage variables was significantly associated with changes in the symptoms in the negative affect domain. In comparison to those who never had formal education, older women with a formal education of 1–6 years or 10 years and above reported a significantly lower level of depressive symptoms at initial measurement (β = −1.21, *p* < 0.001 and β = −2.22, *p* < 0.001 respectively). Having no full time job was associated with a higher level of depressive symptoms than housekeepers (β = 0.70, *p* < 0.01) at initial measurement. Women who experienced economic strain in mid-life (β = 1.98, *p* < 0.001) demonstrated a higher level of depressive symptoms than their counterparts. Only positive perception of social support in caring showed significant impact on the depressive symptomatology, with lower levels of depressive symptoms initially (β = −1.74, *p* <0.01) and a greater change over time as respondents aged (β = 0.10, p <0.05).

Unlike the negative affect domain, educational attainment did not have a significant effect initially in the lack of positive affect domain. Women with 7–9 years of education had a significantly steeper decrease in level of symptoms in the lack of positive affect than those who had no formal education as they became older (β = −0.05, *p* < 0.05). As in the negative affect domain, economic strain in mid-life (β = 0.89, *p* < 0.001) had a deteriorating effect on depressive trajectory at initial measurement. Lower levels of lack of positive affect were associated with positive social support in caring (β = −0.67, *p* < 0.05), and sick care (β = −0.63, *p* < 0.05). No significant effect was found for the interaction of perceived social support and age.

## Discussion

Results of this study suggested mid-life socioeconomic disadvantage and perceived social support in caring and sick care inhibited the onset of depressive trajectories among older Taiwanese women. In contrast, only perceived social support in caring was associated with subsequent increased levels of depressive symptomatology. Our analysis illustrated the multidimensional nature of this relationship; that is, educational attainment, employment status and lack of economic strain inhibited the onset of depressive symptomatology. However, mid-life occupation showed no impact on older Taiwanese women’s mental health.

The main purpose of this study was to determine whether Taiwanese females in different social economic situations have different depressive trajectories as they grow older. Economic strain was found to have an extremely significant impact on the trajectories in both domains, showing the importance of subjective SES on depressive symptomatology among Taiwanese women. This result was consistent with previous findings [22]. Other mid-life SES factors were also found to have some impact on the trajectory of the negative affect domain, while there was no significant impact on the trajectory for lack of positive affect. The findings once again suggested multidimensional indicators of SES should be taken into consideration to increase understanding of the longitudinal relationships between SES and depressive symptomatology. Different SES indicators in depressive symptomatology may require different strategies through which mid-life SES could affect the development of depressive symptoms in later life.

Our results further substantiated the body of research showing the benefit of education for women, as demonstrated by lower initial levels of depressive symptoms for women who had formal education versus women who did not have formal education [15,21]. The gap increased as higher levels of education were compared. In addition, our analyses indicated a significant effect of education on slowing changes over time in depressive symptoms among women with 7–9 years of education in compared to those who had no formal education as they became older. As suggested by the life-course hypothesis, people in lower SES have limited socioeconomic resources, which, in turn, lead to poorer long-term health [18]. Our findings supported the “accumulation effect” [10] that gaps in depressive symptomatology clearly increased over time between women with no education and those with 7–9 years of education. This deteriorating effect continued for the socioeconomic disadvantaged even in their later years. Above finding was consistent with the findings of Singh-Manoux et al. [14]. However, life time major occupation had no significant impact on the trajectory in both domains. This may be partly due to the fact that a woman’s occupation represents not only personal abilities but social expectation [34]. Women were much more likely than men to retreat from the labor market during their reproductive years, particularly in East-Asian societies. Future studies on this issue will need a category scheme that is far more subtle and complete than the one used here and in the Taiwan study. In sum, the findings of this study paralleled previous western research that suggested the importance of educational attainment as an indicator of SES for women [35].

Given about one-third of the sample regarded themselves as housekeepers, we retained this category regarding employment status. We found women who did not have a full-time job in mid-life but rather served as housekeeper had higher initial levels of depressive symptoms, however, the difference in initial status between those who had a full-time job and served as a housekeeper was not significant. Boye [36] had found time spent in fulltime employment or housework was associated with lower depression level, which partly resembled our findings [36]. To understand the actual impact of employment status on depression to a greater degree, more research is needed, especially for older women.

Perceived social support in caring and sick care was associated with initial lower levels of depressive symptoms for two domains among older women. Our results suggested the long-term positive effect of perceived support toward depressive symptoms in later life. These results expanded our previous understanding of social support as a cross-sectional effect. In addition, our analysis suggested women with a positive perception of social support in caring and sick care reported fewer depressive symptoms. Women with a positive perception of social support may be more motivated to seek social interactions and participation. As social capital theories suggest, social interaction can provide individuals with an expanded social network that results in more sources of interpersonal support, increased contact with social resources and even healthcare services that promote greater well-being [37,38].

Previous research distinguished the moderating effect of social support between disability and depression [39] among elders. To avoid possible confounding effects and present our results in a simplified way, we held health-related variables in abeyance in this study for two reasons: (1) our sample was much younger than previous samples showing little variations in physical functions; and (2) the advantage of using growth curve models in which change is modeled on the basis of aging that takes physical decline into consideration. We have separately run the models with physical health measures. Similar major findings were obtained. Furthermore, this study found SES and perceived social support in mid-life to be independent of one another regarding impact on depressive symptoms. This result was similar to previous findings [40] in which SES was not shown to influence social support for women.

Using growth curve models avoided the possible bias associated with the dependence on repeated measures in longitudinal data. This analytical method has greater tolerance for missing data, maximizes sample size and thereby retains the diversity of sample characteristics. Moreover, growth curve models detect the impact of both initial status and changes of SES and perceived support in mid-life in the depressive trajectories simultaneously [41]. However, this work is not without limitations. First, we excluded non-self-respondents, because the outcome and explanatory variables are based on self-reports. This exclusion may have yielded an analytical sample that is “healthier”, and perhaps introduces a “health effect”. Second, the TLSA data were based on self-reported recall of mid-life SES and depressive symptoms, raising the issue of recall bias. Third, while previous studies have suggested the importance of self-esteem and coping strategies in depressive symptomatology [23], such data were not available for the present investigation. Fourth, the scope of this study was limited to socioeconomic disadvantage and social support of women and its effects on trajectories of depressive symptomatology. As the Taiwanese place a high value on family, we have conducted separate analyses in which we have included education and main occupation of women’s partners. Although the SES of the partners did not produce an independent effect on women’s depressive symptomatology in our investigation, future research is needed to specifically compare the influence SES and social support of women vs. their partners over life-course. Fifth, SES and social support used in the analyses are based on mid-life measures. Prior research indicated these variables were likely to have changed [15]. Analysis of the additional time-varying covariates was beyond the scope of this investigation which focused on the time-varying nature of SES and social support as well as the relationship of these focal constructs with mental health. The next logical step in this line of inquiry is to investigate the role of changes in SES and social support experienced by women in the pathway between SES, social support, and emotional health.

## Conclusions

In summary, our findings demonstrated the multi-dimensional nature of SES and perceived social support and their effects on trajectories of depressive symptomatology within two distinct domains for older Taiwanese women. These results underscore the importance of mid-life social environment on depressive symptomatology in the population of elderly women, independent of aging. Given rapid social change, this longitudinal analysis with a cohort sample provided important information about unique cohort experiences in this research area among these women in Taiwan. To our knowledge, this might be the first research in Asia that used longitudinal data to explore SES differences as well as perceived social supports in relation to trajectories of depressive symptomatology among community-based older women. The findings suggested health professionals developing programs and messages to promote mental health among older women should consider the impact of mid-life SES and perceived social support in their design.

### Note

TLSA data can be applied for research use by approval of the Administration of Health Promotion at Ministry of Health and Welfare in Taiwan.

## Competing interests

The authors declare that they have no competing interests.

## Authors’ contributions

YYC was responsible for development of study hypotheses, data analysis, and drafting of the article. CC contributed to developing study hypotheses, critical revision, and finalizing of the article. KK contributed to critical revision of the article. All authors involved in the writing of the paper, and all approved the final submission.

## Pre-publication history

The pre-publication history for this paper can be accessed here:

http://www.biomedcentral.com/1471-2458/14/384/prepub
